# Perceptions of team-based learning using the Team-Based Learning Student Assessment Instrument: an exploratory analysis amongst pharmacy and biomedical students in the United Kingdom

**DOI:** 10.3352/jeehp.2019.16.23

**Published:** 2019-08-21

**Authors:** Prabha Parthasarathy, Bugewa Apampa, Andrea Manfrin

**Affiliations:** 1 Faculty of Medicine, Imperial College, London, UK; 2 Sussex Pharmacy, School of Life Sciences, University of Sussex, Falmer, UK; Hallym University, Korea

**Keywords:** Cohort studies, Pharmacies, Self-directed learning as topic, Students, United Kingdom

## Abstract

**Purpose:**

This study aimed to evaluate students’ perception of team-based learning (TBL) amongst a cohort exposed to this methodology for the first time at a university in the United Kingdom.

**Methods:**

Between November and December 2018, 26 first-year Master of Pharmacy and 90 second-year Biomedical Science students of the School of Life Sciences, University of Sussex, United Kingdom were invited to participate and requested to complete a questionnaire that contained quantitative and qualitative questions. The quantitative component was based on the Team-Based Learning Student Assessment Instrument (TBL-SAI). It additionally contained questions about key student characteristics.

**Results:**

The response rate was 60% (70 of 116); of the participants, 74% (n=52) were females and 26% (n=18) males. The percentage of agreement in the TBL-SAI suggested a favourable response to TBL. The overall mean score for the TBL-SAI was 115.6 (standard deviation, 5.6; maximum score, 140), which was above the threshold of 102, thus suggesting a preference for TBL. Statistically significant differences were not found according to demographic characteristics. Students who predicted a final grade of ≥70% strongly agreed that TBL helped improve their grades. Some students highlighted issues with working in teams, and only 56% of students agreed that they could learn better in a team setting.

**Conclusion:**

This study shows that students exposed to TBL for the first time favoured several aspects of TBL. However, more focused strategies including team-building activities and expert facilitation skills could potentially tackle resistance to working in teams.

## Introduction

Team-based learning (TBL) was introduced in the United States as an alternative to traditional methods of teaching by Larry Michaelsen, and since then it has become an increasingly popular teaching strategy worldwide [1]. A systematic literature review documented the use of TBL in the health professions in 23 countries, demonstrating that the number of articles published on TBL increased steadily, tripling between 2011 and 2016 [2]. There is some published literature from the United Kingdom (UK) [3-5]; however, literature on this topic from the UK remains scant in comparison to the global landscape, and hence there is a need to define its use and perception in the context of local demographics.

Starting in 2016, at the University of Sussex, we implemented TBL in a blended learning environment, supplementing and partly replacing lectures as the teaching method across 2 different degrees: pharmacy and biomedical science. In the UK, students are generally accustomed to didactic teaching; TBL represents a shift to dialogic learning, which leads to new understanding and knowledge, greater student engagement, and improved thinking skills because meanings are constructed from the inside by learners in a dialogue instead of being imposed from outside. The aim of the study was to evaluate students’ perception of TBL amongst a cohort of students who were exposed to this methodology for the first time at this university with the aim of informing future curriculum development. The hypothesis was to assess whether students favoured TBL over lectures.

## Methods

### Ethics statement

The study was conducted in accordance with the Helsinki Declaration of 1975 as revised in 2008, and received ethical approval from the Life-Sciences-Psychology Cluster-based Research Ethics Committee of the University of Sussex on 9/11/2018 (ref: ER/PP225/1). Informed consent was obtained from all individual participants included in the study.

All data were handled following the requirements of the Data Protection Act (2018) and/or the General Data Protection Regulation (GDPR) 2016 according to European Union law; therefore, data were anonymised and stripped of any identifiable references to the participants.

### Study design

This was a cohort study.

### Study population

In this study, 26 first-year pharmacy and 90 second-year biomedical science students were invited to participate.

TBL sessions in the pharmacy programme were embedded into the MPharm curriculum and conducted every 2 weeks in a 2-hour teaching session. Pre-reading consisted of specific lecture content and self-directed learning through books and internet resources. In a 2-hour session, students completed the readiness assurance test (RAT) and application exercises. In contrast to pharmacy class, biomedical science students were exposed to TBL for the first time in the second year in a module covering general anatomy and haematology. The RAT sessions were based around pre-reading in the form of mini-PowerPoint recorded lectures. This was followed by 1–4 hours’ lecture on each theme and subsequently, application exercises integrating the RAT and lecture content. Both courses had 5 TBL sessions in the 12-week teaching term.

### Research instrument

The validated Team-Based Learning Student Assessment Instrument (TBL-SAI) developed by Mennenga was used [6]. The questionnaire was adopted to gather also students’ characteristics such as age, gender, ethnicity, residence, status, entry qualification to the university, and prediction of their grade for the module. Permission for using TBL-SAI was obtained from the original author. The TBL-SAI includes 33 questions aimed at investigating 3 dimensions: accountability (8 items), preference for lecture or TBL (16 items) and student satisfaction (9 items) with a 5-point Likert scale. During the final TBL teaching session, and following informed consent, students were invited to fill out an online questionnaire delivered through a web platform called Qualtrics available from: https://www.qualtrics.com.

### Sample size calculation

The required sample size (n=67) was calculated using the t-test to assess the mean difference from the constant (1 sample) with 2 tails, effect size (Cohen d=0.5), α=0.05, critical t=±1.99, and a power of 98%. The power analysis was conducted using G*Power ver. 3.1.9.3 (http://www.gpower.hhu.de/) [7].

### Statistical analysis

Quantitative data were analysed using descriptive statistics. The internal consistency of TBL-SAI was assessed using the Cronbach α. Furthermore, a scoring system was applied to TBL-SAI, according to Nation et al. [4], which defined a score of >102 as indicative of a preference for TBL. The Kolmogorov-Smirnov test was used to assess the normality of the data distribution. Data were presented as range, mean and standard deviation as suggested by Norman (2010) [8]. The Student t-test was used for analysing demographic characteristics and the mean values of the total TBL-SAI score. Differences in the mean of the total TBL-SAI score (as thresholds) according to demographic characteristics were analysed using the Pearson chi-square (χ^2^) and/or Fisher exact tests, with odds ratios and 95% confidence intervals.

Correspondence analysis, a multivariate statistical technique, was conducted to identify the relationships between 2 categorical variables, the impact of TBL on grades and grade prediction. Qualitative comments were analysed using thematic analysis, stratified as reflective of positive or negative attributes. Negative comments were further classified into themes such as issues with working in teams and the conduct of TBL activities. Qualitative comments were linked to the total score obtained with the TBL-SAI using the mean value as a threshold. The analyses were conducted using IBM SPSS ver. 24.0 (IBM Corp., Armonk, NY, USA) and Microsoft Excel ver. 2016 (Microsoft Corp., Redmond, WA, USA). A P-value <0.05 was considered to indicate statistical significance.

## Results

### Demographic characteristics

Seventy students participated in the study. The response rate was 60.3% (70 of 116); this included 52 (74.3%) biomedical science students and 18 (25.7%) pharmacy students, giving response rates of 57.7% (52 of 90) and 69.2% (18 of 26), respectively. There was a predominance of female students, and 90% of the students in our samples were in the 16- to 24-year range (Table 1).

### Internal consistency of the TBL-SAI

The Cronbach α for the TBL-SAI (33 items) was 0.651; this low value was accepted due to the exploratory nature of the analysis, which is in line with the study conducted by Jeno et al. [9] in 2017. The Cronbach α values for accountability, preference for lecture or TBL, and student satisfaction were 0.501, 0.412, and 0.626, respectively.

### Students’ responses to the TBL-SAI instrument

**Fig. 1.**
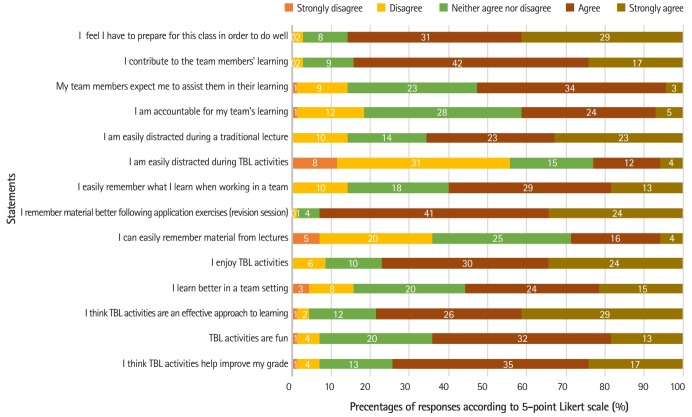
Students’ responses to selected statements across 3 domains: accountability, preference, and student satisfaction. Numbers within bars represent the frequency of responses for each option of the 5-point Likert scale. TBL, team-based learning.

Fig. 1 presents students’ responses to selected statements (14 of 33) across 3 domains. The responses to most questions suggested that students favourably accepted several features of TBL. A high percentage of students agreed and strongly agreed that they felt the need to prepare for the class (85.7%) and contribute to their team’s learning (84.2%). TBL appeared to be more engaging, as more students agreed that they were easily distracted in lectures as compared to TBL (65.7% versus 22.9%). It was also a useful revision tool, with more students agreeing that they found it easier to remember material following TBL than after lectures (74.2% versus 28.6%); in particular, strong agreement was noted with the statement “I remember material easier following application exercises” (92.9%). Overall, a high percentage of students agreed that they had a good experience with TBL (77.1%) and that it was fun (64.2%).

While most elements of TBL were positively received by students, we noted some resistance, which could be gathered from a lower percentage agreeing that they felt accountable for the team’s learning (41.4%) and had the perception of learning better in a team (55.7%). The responses for each domain of the TBL-SAI are presented in Appendix 1.

All 3 dimensions included in the TBL-SIA instrument achieved a score well above the threshold favouring TBL (Table 2).

### Demographic characteristics and TBL-SAI scores

Table 3 shows that statistically significant differences were not identified when computing demographic characteristics and the mean total TBL-SAI total score. A further analysis was conducted of demographic characteristics according to the total score threshold (102) and total mean score threshold (115.6), and this analysis likewise did not show statistically significant differences (Table 4).

### Correspondence analysis

**Fig. 2.**
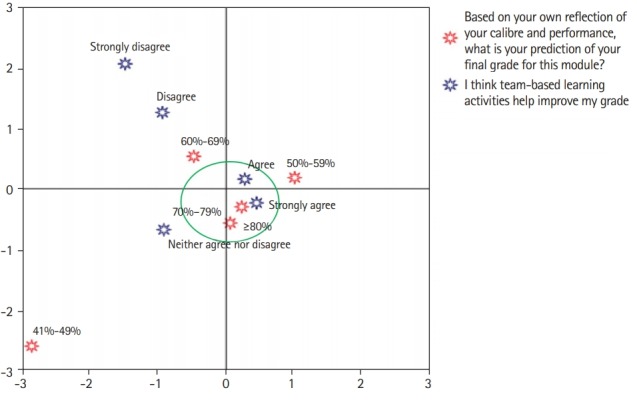
Correspondence analysis between grade prediction and team-based learning activities. Correspondence analysis is a statistical technique that graphically display a 2-way table by reproducing the distances between the row or column coordinates (patterns of relative frequencies across the columns or rows, respectively) in a low-dimensional solution. It measures the associations amongst variables. The closer are the red and blue stars on the plane the higher will be the association (e.g., strongly agree and 70%–79% ). The red and blue stars within the green circle are highly associated.

Most students predicted a grade in the range of 60%–69% (n=25, 35.7%) or 70%–79% (n=33, 47.1%). Due to the small numbers in the other grade categories, conclusions could not be drawn for them. The analysis suggested a link between students who predicted a grade of 70% or above and those who agreed with the statement “I think team-based learning activities help improve my grade” (Fig. 2).

### Students’ comments

Amongst 70 respondents, 24 provided qualitative comments, which were stratified and grouped according to the 3 themes presented in Table 5. Out of 24 students, 18 students highlighted at least 1 positive attribute of TBL, including comments such as:

“TBL should be incorporated into all modules.” (Biomedical science student no. 15)
“I enjoyed working in a team, especially as it is a nice break from the monotony of most non-interactive lectures.” (Biomedical science student no. 48)
“I prefer this over lectures.” (Biomedical science student no. 14)
“TBL is a great way to make a class more interesting and therefore it gives me greater stamina to learn.” (Pharmacy student no. 65)

Six students highlighted issues with working in teams, and 5 of these comments were from students with TBL-SAI scores of less than 115.6. However, all these students still had a total score >102, which is the defined threshold for a positive perception of TBL in the TBL-SAI instrument. Comments included:

“Sometimes working with people you’re not friends with makes team-based learning less effective and a little isolating.” (Biomedical science student no. 11)
“I would have preferred smaller groups too, as it is sometimes intimidating working in a big group of people I don’t know in such academic setting.” (Biomedical science student no. 16).

All comments highlighting this issue around teamwork were from biomedical science students. In addition, 6 students across biomedical science and pharmacy highlighted issues related to the actual conduct of TBL, with key issues being the lack of recorded material for the individual RAT and team RAT activities, the time allocated for discussion, and the length and timing of application exercises. The raw data are available in Supplement 1.

## Discussion

The main aim of our study was to explore students’ perceptions of TBL when they were exposed to this methodology for the first time at university. We surveyed students across courses in 2 different subject areas: pharmacy and biomedical science. Although there were inherent differences in the blended approach in these courses, including the year of study when TBL was first introduced (first year in pharmacy and second year in biomedical science), we believe this did not have an impact on the overall perceptions, which appeared to be favourable for TBL. The overall TBL-SAI score of 116 was higher than the threshold (≥102) that suggests a preference for TBL, and similar scores were noted in other similar healthcare-related subject areas [4,10,11]. Statistically significant differences were not found in the total TBL-SAI mean scores and thresholds (102 versus 115.6) according to demographic characteristics and disciplines.

Several key pedagogical ideologies underpin TBL. These include encouraging self-reading, holding students accountable for their learning, and the use of effective authentic assignments that trigger discussions amongst teams [12]. The percentage of agreement with several statements suggests that our courses were successful in embedding these attributes into the learning process. Over two-thirds of the students agreed that they needed to contribute to their team’s learning, and the majority felt that they had to prepare for class if they would like to do well. Students found TBL more engaging and less distracting than lectures.

In our study, correspondence analysis showed a link between perceived academic potential (shown by anticipated grades of 70% and above) and agreement with the statement “I think team-based learning activities help improve my grade.” Improved perceptions amongst high academic achievers have been documented by other studies [13,14]. Vasan et al. [13] noted a similar pattern and suggested that high achievers may have more effective existing study patterns, such as self-study and group study. It is also possible that team dynamics and interactions may alter perceptions, as academically more able students have the chance to dominate academic discussions and thus favourably perceive TBL. At this stage, it would be premature to draw conclusions. The use of qualitative methods such as focus groups or interviews could reveal reasons behind grade perceptions and inform future sessions.

Although the responses to the TBL-SAI suggested a preference for TBL, we noted a few challenges with TBL. Several students’ comments indicated team-related issues, which appeared to drive a lower score in the TBL-SAI instrument. The specific comments emanating from the biomedical science students could be attributed to large class sizes, a sudden resistance in the second year when students were being forced to shake off some habits that were formed in the first year, and the sessions being facilitated by a single instructor. We also hypothesised that students’ characteristics such as age or gender could influence this perception, however, we did not find any statistically significant differences according to age or gender. In addition, only 56% agreed or strongly agreed that they learned better in a team setting, and this was similar in both courses. Sharma et al. [10] in 2017 also noted resistance as gauged by responses to the relevant TBL-SAI questions. Resistance towards teamwork could be the result of instructors’ lack of experience with TBL. Davidson [15] in 2011 found a stronger preference for active learning in a sequential cohort of medical students, which was attributed to the increasing experience of educators with active learning strategies. Resistance towards teamwork could be tackled by structured team-building exercises, designing discussion-provoking exercises, and expert facilitation skills. Teamwork could also be improved if teams were together longer than 12 weeks; as they appeared to have remained in the phase of storming/forming rather than performing, which might explain students’ resistance.

Our study had some limitations. The number of participants varied across both surveyed courses and in addition, we had a small sample size. Based on our findings, we believe it is important to conduct a larger study to confirm our findings and establish any associations with specific student characteristics. This, in turn, could inform the way teams are formed, which could be crucial for effective team functioning. In addition, it would be useful to determine whether perceptions of teamwork change as students gain more exposure to TBL and to evaluate the impact of specific strategies on the perception of teamwork.

In conclusion, our study showed that students who were exposed to TBL as a novel teaching strategy for the first time favoured it over lectures and found it engaging. Thus, TBL is a strong pedagogical tool that can be used to promote enthusiasm towards learning and to supplement lectures. Resistance to teamwork needs to be tacked by strategies that promote team discussions, which has the potential of enhancing the learning experience.

## Figures and Tables

**Table 1.** Demographic characteristics of participants

Students’ demographics	No. (%)
Gender	
Female	52 (74.3)
Male	18 (25.7)
Age range (yr)	
16–24	63 (90.0)
>24	7 (10.0)
Ethnicity	
White	44 (62.9)
Others	26 (37.1)
Residence status	
UK/EU	67 (95.7)
Non-UK/Non-EU	3 (4.3)
Entry qualification	
A level^a)^/IB^b)^	41 (58.6)
Others	29 (41.4)

UK, United Kingdom; EU, European Union.

a) Advanced level, or A level, is a main school-leaving qualification in England, Wales, Northern Ireland, the Channel Islands, and the Isle of Man.

b) International Baccalaureate is an international educational qualification.

**Table 2.** Scores according to the dimensions of the TBL-SAI questionnaire

	Accountability	Preference of teaching style	Students’ satisfaction	Total score
Reference range	Scores >25 favour TBL	Scores >45 favour TBL	Scores >28 favour TBL	Scores >102 favour TBL
Range	20–35	43–69	22–39	94–140
Mean±SD	28.6±3.1	55.2±4.9	31.8±4.1	115.6±5.6

The reference range represents the threshold for each dimension and the total score of TBL-SAI. The measures of the lowest and highest TBL-SAI scores are represented by the range, central tendency by the mean, and dispersion by the SD. TBL-SAI, Team-Based Learning Student Assessment Instrument; TBL, team-based learning; SD, standard deviation.

**Table 3.** Demographic characteristics according to the means of the total TBL-SAI score

Demographic characteristic	Total TBL-SAI score		95% Confidence interval	P-value^a)^
	Mean	Mean difference		
Gender				
Male	114.05	-2.11	-6.800 to 2.565	0.370
Female	116.17			
Age range (yr)				
16–24	115.09	-5.33	-12.074 to 1.407	0.119
>24	120.42			
Ethnicity				
White	114.38	-3.34	-7.528 to 0.839	0.115
Other	117.73			
Entry qualification				
A level^b)^/IB^c)^	116.39	1.83	-2.317 to 5.994	0.318
Other qualifications	114.55			
Degree				
Biomedical science	116.03	1.59	-3.101 to 6.289	0.500
Pharmacy	114.44			

TBL-SAI, Team-Based Learning Student Assessment Instrument.

a) Student t-test; statistical significance P<0.05. Residence was not included in the analysis due to the very low numbers of non-United Kingdom/non-European Union students (n=3).

b) Advanced level, or A level, is a main school-leaving qualification in England, Wales, Northern Ireland, the Channel Islands, and the Isle of Man.

c) International Baccalaureate is an international educational qualification.

**Table 4.** Demographic characteristics and total TBL-SAI score thresholds

Demographic characteristic	TBL^a)^				TBL mean^b)^			
	≤102	>102	OR (95% CI)	P-value^c)^	≤115.6	>115.6	OR (95% CI)	P-value
Gender								
Male	2 (11.1)	16 (88.9)	2.042 (0.313–13.328)	0.597	10 (55.6)	8 (44.4)	0.991 (0.337–2.916)	0.987
Female	3 (5.8)	49 (94.2)			29 (55.8)	23 (44.2)		
Age range (yr)								
16–24	4 (6.3)	59 (93.7)	0.4 (0.039–4.251)	0.419	36 (57.1)	27 (42.9)	1.778 (0.367–8.613)	0.692
>24	1 (14.3)	69 (85.7)			3 (342.9)	4 (57.1)		
Ethnicity								
White	3 (6.8)	41 (93.2)	0.878 (0.137–5.633)	1	27 (61.4)	17 (38.6)	1.853 (0.695–4.943)	0.216
Others	2 (7.7)	24 (92.3)			12 (46.2)	14 (53.8)		
Entry qualification								
A level^d)^/IB^e)^	2 (4.9)	39 (95.1)	0.444 (0.069–2.846)	0.642	23 (56.1)	18 (43.9)	1.038 (0.399–2.704)	0.939
Other qualifications	3 (10.3)	26 (89.7)			16 (55.2)	13 (44.8)		
Degree								
Biomedical science	3 (5.8)	49 (94.2)	0.49 (0.075–3.197)	0.597	27 (51.9)	25 (48.1)	0.54 (0.176–1.656)	0.278
Pharmacy	2 (11.1)	16 (88.9)			12 (66.7)	6 (33.3)		

Values are presented as number (%) or OR (95% CI), unless otherwise stated. TBL-SAI, Team-Based Learning Student Assessment Instrument; TBL, team-based learning; OR, odds ratio; CI, confidence interval; FET, Fisher exact test.

a) TBL cut-off score threshold=102.

b) Mean of the total score as threshold=115.6.

c) By Pearson chi-square or FET; The choice between χ2 and FET was made according to the data requirements.

d) Advanced level, or A level, is a main school-leaving qualification in England, Wales, Northern Ireland, the Channel Islands, and the Isle of Man.

e) International Baccalaureate is an international educational qualification.

**Table 5.** Themes according to the mean of the total TBL-SAI score as the threshold

Themes	Score >115.6 (n=17)	Score ≤115.6 (n=7)
Positive features of TBL highlighted	12	6
Team issues highlighted	1	5
Conduct issues highlighted	4	2

TBL-SAI, Team-Based Learning Student Assessment Instrument; TBL, team-based learning.

## Supplementary materials

Supplement 1. Data files are available from https://doi.org/10.7910/DVN/LNW5MK

Supplement 2. Audio recording of the abstract.

## Appendix 1. The results of the TBL-SAI for accountability, preference for TBL over lectures, and student satisfaction, respectively

Numbers within bars represent the frequency of responses for each option of the 5-point Likert scale. TBL-SAI, Team-Based Learning Student Assessment Instrument; TBL, team-based learning.

Numbers within bars represent the frequency of responses for each option of the 5-point Likert scale. TBL, team-based learning.

Numbers within bars represent the frequency of responses for each option of the 5-point Likert scale. TBL, team-based learning.
